# Acidification is an Essential Process of Cold Atmospheric Plasma and Promotes the Anti-Cancer Effect on Malignant Melanoma Cells

**DOI:** 10.3390/cancers11050671

**Published:** 2019-05-14

**Authors:** Christin Schneider, Lisa Gebhardt, Stephanie Arndt, Sigrid Karrer, Julia L. Zimmermann, Michael J. M. Fischer, Anja-Katrin Bosserhoff

**Affiliations:** 1 Institute of Biochemistry, Emil-Fischer-Center, University of Erlangen-Nürnberg, 91054 Erlangen, Germany; christin.schneider@fau.de; 2 Institute of Physiology and Pathophysiology, University of Erlangen-Nürnberg, 91054 Erlangen, Germany; lisa.gebhardt@fau.de (L.G.); michael.jm.fischer@meduniwien.ac.at (M.J.M.F.); 3 Department of Dermatology, University Hospital Regensburg, 93053 Regensburg, Germany; stephanie.arndt@ukr.de (S.A.); sigrid.karrer@ukr.de (S.K.); 4 Terraplasma medical GmbH, 85748 Garching, Germany; zimmermann@terraplasm.com; 5 Institute of Physiology, Medical University of Vienna, 1090 Vienna, Austria; 6 Comprehensive Cancer Center (CCC) Erlangen-EMN, 91054 Erlangen, Germany

**Keywords:** cold atmospheric plasma, malignant melanoma, calcium signaling, acidification, nitration

## Abstract

(1) Background: Cold atmospheric plasma (CAP) is ionized gas near room temperature. The anti-cancer effects of CAP were confirmed for several cancer types and were attributed to CAP-induced reactive species. However, the mode of action of CAP is still not well understood. (2) Methods: Changes in cytoplasmic Ca^2+^ level after CAP treatment of malignant melanoma cells were analyzed via the intracellular Ca^2+^ indicator fura-2 AM. CAP-produced reactive species were determined by fluorescence spectroscopic and protein nitration by Western Blot analysis. (3) Results: CAP caused a strong acidification of water and solutions that were buffered with the so-called Good buffers, while phosphate-buffered solutions with higher buffer capacity showed minor pH reductions. The CAP-induced Ca^2+^ influx in melanoma cells was stronger in acidic pH than in physiological conditions. NO formation that is induced by CAP was dose- and pH-dependent and CAP-treated solutions only caused protein nitration in cells under acidic conditions. (4) Conclusions: We describe the impact of CAP-induced acidification on the anti-cancer effects of CAP. A synergistic effect of CAP-induced ROS, RNS, and acidic conditions affected the intracellular Ca^2+^ level of melanoma cells. As the microenvironment of tumors is often acidic, further acidification might be one reason for the specific anti-cancer effects of CAP.

## 1. Introduction

Cold atmospheric plasma (CAP) is ionized gas near room temperature, which is produced at an atmospheric pressure. It consists of reactive oxygen (ROS) and reactive nitrogen (RNS) species, charged particles, and an optical emission also in the UV range [1]. It is well known that CAP shows antibacterial, antiviral, and antifungal effects, and it can therefore be used for disinfection or sterilization of, for instance, hands or surgical instruments [2,3,4,5,6]. CAP can promote the healing of chronic wounds due to its antibacterial activity [7] and by inducing angiogenesis and wound healing relevant molecules, like interleukin 8 [8,9,10]. CAP has the potential to be a new promising therapy for several cancer types, including malignant melanoma [11]. Malignant melanoma is one of the most aggressive cancer diseases, because of its high malignancy and therapy resistance. Although it is responsible for only 2% of all skin cancers, over 75% of skin cancer deaths were attributed to a melanoma disease [12]. Hence, there is substantial medical need for the development of new therapeutic approaches.

The most commonly observed effect after CAP treatment of cancer cells was the induction of apoptosis, as provoked by CAP-produced ROS and RNS. In this regard, especially DNA damage and depolarization of mitochondrial membrane potential could be detected [13,14,15,16,17]. Interestingly, CAP-treated solutions, like medium or saline, also displayed clear anti-cancer effects [18,19,20]. One special feature of CAP is its high selectivity against cancer cells [21,22,23]. Moreover, CAP also showed the killing effects on chemo-resistant cancer cells in vitro and in vivo and, in some cases, CAP-induced restoration of chemo-sensitivity was described [14,24,25].

Our group determined dose-dependent effects of CAP on malignant melanoma cells using a surface micro-discharge (SMD) plasma device [13]. In that study, a longer CAP application (2 min) caused DNA damage and apoptosis, while a shorter CAP application (1 min) induced senescence. Recently, we demonstrated that the direct and indirect CAP treatment of melanoma cells causes a Ca^2+^ influx that is derived from intracellular stores, which trigger the induction of CAP-induced senescence [26]. Interestingly, the addition of CAP-treated solution after an incubation time of 1 h also led to a cytoplasmic Ca^2+^ elevation. Thus, we assume that mainly long-lived CAP-produced ROS and RNS are the cause of the CAP-induced Ca^2+^ influx in malignant melanoma cells.

Until today, the molecular background of CAP on cancer cells and the exact involved species are not well understood. Moreover, a comparison of plasma effects is hardly possible, because of the use of distinct plasma sources resulting in different plasma components [27]. The purpose of this study was to narrow down the reactive species that are involved in CAP-induced Ca^2+^ influx, and thus, to get a deeper understanding of the mode of action of CAP on cancer cells.

## 2. Results

### 2.1. Effects of Different Solutions on CAP-Induced Ca^2+^ Influx

**Figure 1:**
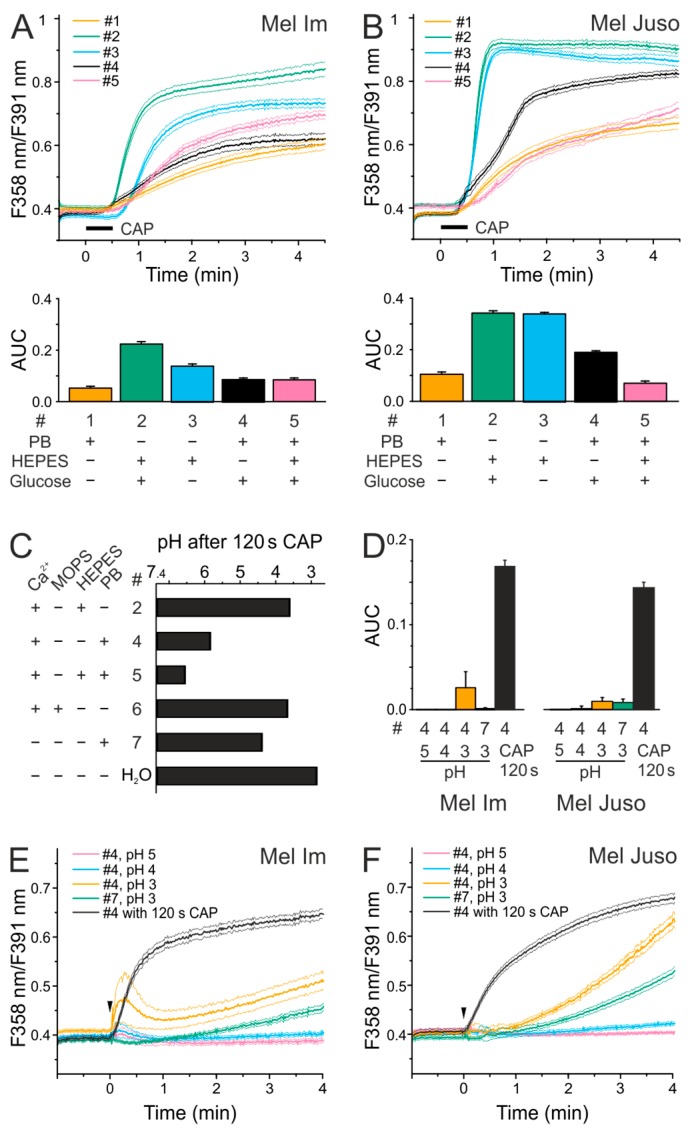
The intensity of cold atmospheric plasma (CAP)-induced Ca^2+^ influx depends on the composition of the solutions and CAP causes an acidification. (**A**,**B**) Analysis of the effect of different solutions on 30 s CAP-induced changes in cytoplasmic Ca^2+^ level of melanoma cells. (**A**) Mel Im (*n* = 137–660) and (**B**) Mel Juso (*n* = 86–500) cells were washed in the Ca^2+^-containing solutions 1, 2, 3, 4, or 5 (#4 data identical to Figure 1 in [26]). After the removal of the respective solutions, cells were treated with 30 s CAP. Traces represent mean and the 99% confidence interval (CI) of the mean. (**C**) The solutions #2, 4, 5, 6, 7 or bidestilled water (pH 7.4) were exposed to 120 s CAP as 7 × 20 µL drops. Measurement of pH was performed in a 1.5 mL reaction vessel. Data are shown as mean. (**D**–**F**). Analysis of the effect of an acidic pH on cytoplasmic Ca^2+^ levels of Mel Im ((**D**,**E**), *n* = 485–999) and Mel Juso ((**D**,**F**), *n* = 275–376) cells. 100 µL of pbECS (#4; pH 3,4,5) and pbECS − Ca^2+^ (#7; pH 3) was added onto melanoma cells (indicated by an arrow). The application of 100 µL of 120 s CAP-treated pbECS (#4) to Mel Im ((**D**,**E**), *n* = 485) and Mel Juso ((**D**,**F**), *n* = 321) cells served as control. Phosphate-buffered (PB), area under the curve (AUC), fluorescence intensity (F).

CAP generates highly reactive molecules. Different physiological buffers were tested, as these differ in buffering capacity as well as in chemical composition, which was assumed to impact the generated molecules. First, we analyzed the impact of different physiological buffer compositions on the CAP-induced Ca^2+^ influx on malignant melanoma cells. Table 1 lists the chemical composition of the applied buffers. Mel Im and Mel Juso cells were washed with the respective solution for 5 min. and changes in the cytoplasmic Ca^2+^ content were measured after 30 s CAP treatment using the intracellular Ca^2+^ indicator fura-2 AM (Figure 1). In all of the tested solutions, CAP-induced a cytosolic Ca^2+^ increase (*p* < 0.001 each, *t*-tests single sample vs. no change). In particular, CAP exposure for 30 s caused a Ca^2+^ influx in both cell lines in phosphate-buffered saline (PBS) (#1), but less than in extracellular solution (ECS) that was buffered with *4-(2-hydroxyethyl)-1-piperazineethanesulfonic acid* HEPES (#2), commonly used for cellular experiments (Mel Im ANOVA, F_(4.2344)_ = 488, *p* < 0.001, honestly significant difference (HSD) post-hoc test, Mel Juso ANOVA, F_(4.1682)_ = 1366, *p* < 0.001, HSD post-hoc test). These solutions mainly differ in their buffer system (HEPES vs. phosphate) and in the presence or absence of glucose. The experiments were performed in ECS HEPES solution with (#2) and without glucose (#3) to investigate a possible dependence on this factor. CAP-induced Ca^2+^ influx in Mel Im cells in the absence of glucose was decreased and the Ca^2+^ response was significantly delayed (*p* < 0.001, HSD post-hoc test). There was no difference between the Ca^2+^ response in the ECS HEPES solution with (#2) or without (#3) glucose in Mel Juso cells (*p* = 0.99, HSD post-hoc test). The Ca^2+^ response in phosphate-buffered extracellular solution (pbECS, #4), as used previously [26], was about half of the response in the ECS HEPES solution (#2, Mel Im and Mel Juso, *p* < 0.001 each, HSD post-hoc test), but higher than in PBS (#1, Mel Im and Mel Juso, *p* < 0.001 each, HSD post-hoc test). The buffer system used had an impact on CAP-induced Ca^2+^ influx, which was either due to differences in production or due to the stability of chemical species. Therefore, we tested the effect of HEPES on CAP-induced Ca^2+^ influx by comparing pbECS solution (#4) with an additionally HEPES-buffered solution (pbECS + HEPES, #5). When compared to the combined buffering, the omission of the phosphate buffering component increased responses (Mel Im and Mel Juso, *p* = 0.001 each, HSD post-hoc tests), but the omission of the HEPES increased responses in Mel Juso (*p* = 0.001, HSD post-hoc test), but not in Mel Im (*p* = 0.99, HSD post-hoc test).

### 2.2. CAP Causes a Decrease in pH but Acidic Solutions Have No Effects on Cytoplasmatic Ca^2+^ Level

As more buffering capacity seemed to reduce the CAP-induced Ca^2+^ influx, we analyzed whether CAP changes the pH value of the solutions. A CAP dose of 120 s decreases the pH of 7.4 of all the solutions tested (*p* < 0.030 each, *t*-tests, Figure 1C). The strongest acidification after CAP treatment was observed in water (H_2_O), resulting in a pH of 3. The solutions that were only buffered with 10 mM of the so-called Good buffers HEPES (#2) or MOPS (#6), showed a pH lower than 4 (Figure 1C). Phosphate-buffered solutions with and without HEPES showed the slightest acidification with pH values of about 6 (pbECS, #4) and 6.5 (pbECS + HEPES, #5). Interestingly, pbECS without Ca^2+^ (#7) resulted in a more acidic pH when compared to the pbECS with Ca^2+^ (#4, *p* = 0.004, *T*-test independent samples).

Next, we asked whether acidic solutions trigger a Ca^2+^ influx in melanoma cells (ANOVA, F_(4.1960)_ = 485 and Mel Juso ANOVA, F_(4.1603)_ = 1340). When compared to the Ca^2+^ increase by a solution exposed to CAP for 120 s, application of acidic solutions caused no biologically relevant Ca^2+^ increase (Mel Im and Mel Juso, *p* < 0.001 each, HSD post-hoc tests, Figure 1D–F). To address this further, the application of pbECS (#4) pH 5 or pH 4 caused no Ca^2+^ increase (n.s.) and pH 3 a slow and gradual increase (marginal as compared to CAP 120 s, 15% in Mel Im and 6% in Mel Juso, *p* < 0.001, *t*-test independent sample, Figure 1E,F). In summary, an acidic pH alone cannot explain the differences in the Ca^2+^ response after CAP treatment.

### 2.3. CAP Causes a Lower Ca^2+^ Influx at Physiological pH Than Under Acidic Conditions

**Figure 2:**
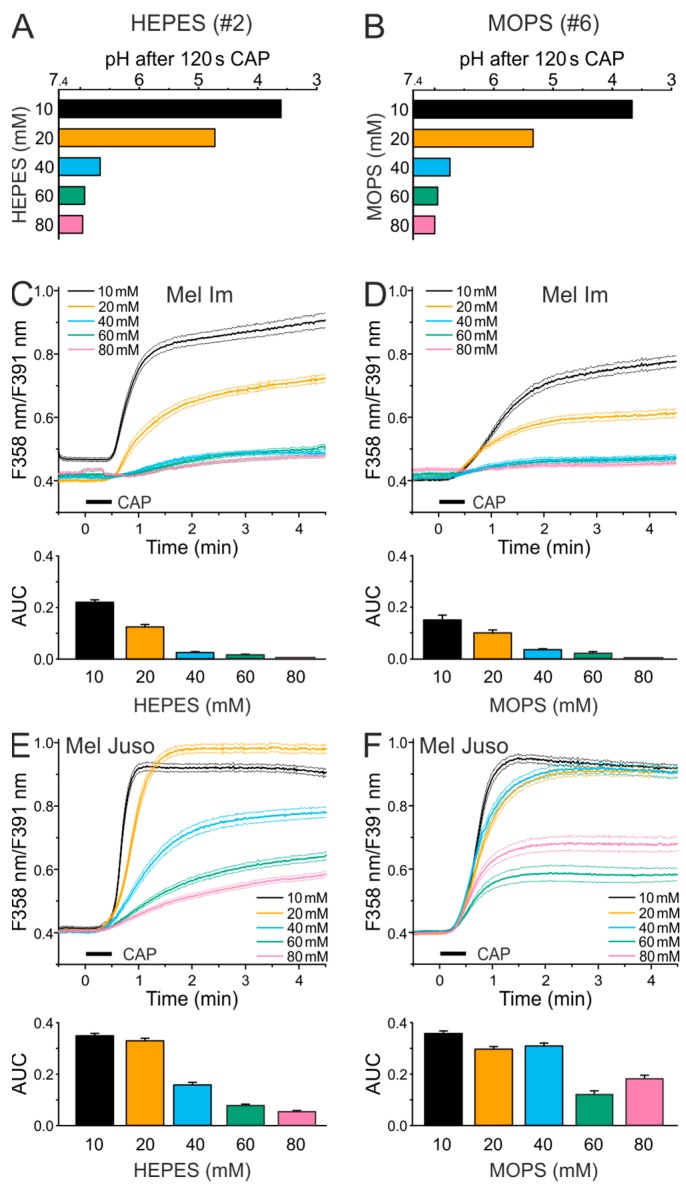
CAP-induced Ca^2+^ influx decreases in Mel Im and Mel Juso cells with increasing buffer concentration. Experiments in HEPES-buffered solutions are reported in the left panels (**A**,**C**,**E**), the respective experiments in MOPS-buffered solutions in the right panels (**B**,**D**,**F**). (**A**,**B**) Measurement of the pH of different HEPES- (**A**) and MOPS- (**B**) buffered solutions (7 × 20 µL) after 120 s CAP treatment, using a 1.5 mL reaction vessel. Data are shown as mean. (**C**–**F**) Cytoplasmic Ca^2+^ levels stimulated by 30 s CAP exposure of Mel Im (*n* = 220–603) and Mel Juso (*n* = 223–377) cells in different HEPES- (**C**,**E**) and MOPS- (**D**,**F**) buffered solutions.

We tested higher buffer concentrations, as the single HEPES- and MOPS-buffered solutions showed the strongest acidification by 120 s of CAP treatment. The CAP-induced acidification decreased with increasing concentration of HEPES (#2, R = 0.97, *p* < 0.001, product-momentum correlation, Figure 2A) and MOPS (#6, R = 0.90, *p* = 0.007, product-momentum correlation, Figure 2B). While the ECS solution with 20 mM HEPES or MOPS had a pH of 4.6 or 5.4 after 120 s CAP, a doubling of the buffer concentration led to a minor CAP-induced pH decrease, with pH values of about 6.6 for both solutions. A buffer concentration of 60 or 80 mM HEPES or MOPS largely abolished the CAP-induced pH reduction. Ca^2+^ imaging experiments with the different ECS HEPES (#2)/MOPS (#6) solutions showed that the Ca^2+^ influx after 30 s CAP decreases with an increasing buffer concentration in Mel Im (*p* < 0.001 each, product-momentum correlations, R = −0.90, −0.96, Figure 2C,D), and Mel Juso (*p* < 0.001 each, product-momentum correlations,−0.93, −0.85, Figure 2E,F). Using 40 mM HEPES or MOPS, the Mel Im cells showed a greatly reduced Ca^2+^ response; whereas, still a marked CAP-induced Ca^2+^ influx was observed in the Mel Juso cells. A threefold increased phosphate amount of the Ca^2+^ free pbECS (#8) solution was required for largely buffering the pH of a 120 s CAP exposure (Figure S1A). In threefold compared to onefold buffer capacity (#8), 30 s CAP treatment caused less Ca^2+^ increase, as also observed in higher HEPES or MOPS buffer concentrations (Mel Im 18% of onefold buffer, Mel Juso 78% of onefold buffer, *p* < 0.001 each, t-test independent samples, Figure S1B,D). However, a substantial fraction of the response remains, even when acidification by CAP is largely buffered.

### 2.4. CAP-produced NOAlone Seems to Have No Effects on CAP-Induced Ca^2+^ Influx

**Figure 3:**
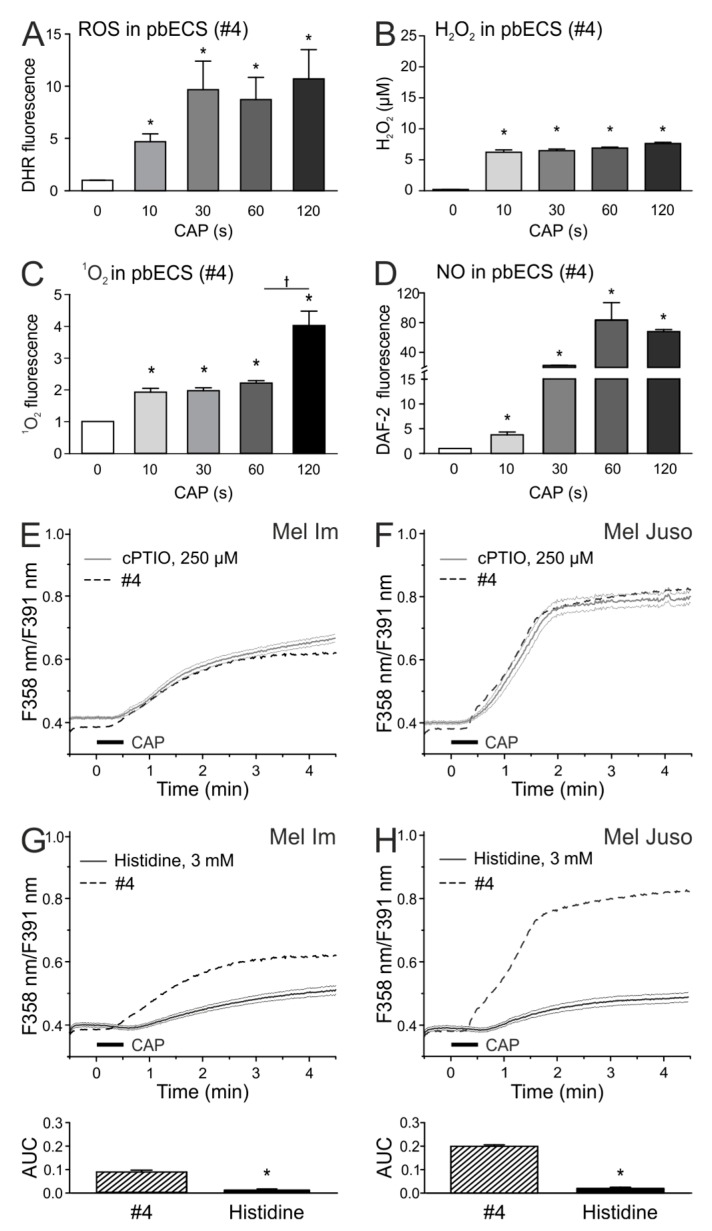
Histidine reduces the Ca^2+^ influx in CAP-treated melanoma cells, nitric oxide (NO) scavenging does not alter the CAP effect. (**A**–**D**) Fluorescence spectroscopic analysis of CAP-induced species production in pbECS (#4; 7 × 20 µL). The fluorescence dyes DHR 123 (**A**), reactive oxygen (ROS)-indicator), Singlet Oxygen Sensor Green Reagent (**C**) and 4,5-diaminofluorescein (DAF-2) (**D**), NO-indicator) were solved in pbECS (#4) at a concentration of 10 µM and exposed to different CAP-doses. (**B**) H_2_O_2_ concentration was determined in duplicates using the Fluorimetic Hydrogen Peroxide Assay Kit. (**E**–**H**). Investigation of the effect of the NO scavenger 2-4-carboxyphenyl-4,4,5,5-tetramethylimidazoline-1-oxyl-3-oxide (cPTIO) (250 µM) and the ROS scavenger histidine (3 µM) on 30 s CAP-induced Ca^2+^ influx in Mel Im ((**E,G**), *n* = 234–470) and Mel Juso ((**F**,**H**), *n* = 84–307) cells. The latter traces are identical to Figure 1 in [26].

Next, we investigated the chemical species that are produced by different CAP exposure times in pbECS. For this purpose, measurements were performed in CAP-exposed droplets. Measurements with the ROS-sensitive dye (DHR 123) showed an increase of fluorescence intensity in CAP-treated pbECS (#4) as compared to the untreated control (*p* < 0.05 each, U-tests, Figure 3A). A H_2_O_2_ production of 6–7 µM was observed in 10 s and longer CAP-treated pbECS (#4) (ANOVA, F_(4.74)_ = 115, *p* < 0.001 each, HSD post-hoc tests, Figure 3B). Using a singlet oxygen (^1^O_2_) reagent, an about twofold higher fluorescence when compared to the control could be observed after 10 s, 30 s, and 60 s CAP (*p* < 0.05 each, U-tests, Figure 3C). Interestingly, 120 s CAP caused a substantial further increase in ^1^O_2_ as compared to the 60 s CAP (marked with a cross, *p* = 0.049, *U*-test, Figure 3C). The strongest CAP duration-dependency in pbECS (#4) could be observed for nitric oxide (NO), which was measured with the NO indicator DAF-2. While 10 s CAP showed an about 5-fold increase of DAF-2 fluorescence, 30 s CAP treatment led to about 20-fold and 60 s to about 80-fold increase NO production in comparison to untreated pbECS (*p* < 0.05 each, *U*-tests, Figure 3D). As the CAP-induced Ca^2+^ influx was shown to increase by treatment time, one or more of the dose-dependently produced species could cause this Ca^2+^ response. Species measurements in Dulbecco’s phosphate buffered saline (DPBS), Dulbecco’s Modified Eagle Medium (DMEM), and RPMI displayed that the composition of the solutions influences the CAP-induced species production (Figure S2). When compared to pbECS, the ROS levels were similar in DPBS, and lower in DMEM and RPMI (ANOVA, F_(3,32)_ = 39.3, *p* = 0.58, *p* < 0.001, and *p* < 0.001, HSD post-hoc tests). When compared to pbECS, H_2_O_2_ levels were similar in DPBS, lower in DMEM and higher in RPMI (*p* = 0.72, *p* < 0.001 and *p* < 0.001, HSD post-hoc tests). As compared to pbECS, the ^1^O_2_ levels were lower in DPBS, higher in DMEM and similar in RPMI (ANOVA, F_(3.32)_ = 61.4, *p* < 0.001, *p* < 0.001, and *p* = 0.99, HSD post-hoc tests). When compared to pbECS, NO levels were lower in all other solutions (ANOVA, F_(3.32)_ = 44.8, *p* < 0.001 each, HSD post-hoc test).

Interestingly, we detected similar ROS as in pbECS in DPBS, but NO production was lower in DPBS. As the CAP-induced Ca^2+^ influx was equally lower in DPBS when compared to pbECS, NO could be important for this effect.

Interestingly, the NO scavenger 2-4-carboxyphenyl-4,4,5,5-tetramethylimidazoline-1-oxyl-3-oxide (cPTIO) had no effects on the CAP-induced Ca^2+^ influx (Figure 3E,F). We further tested the ROS scavenger histidine, which display quenching effects on ^1^O_2_, H_2_O_2_, and •OH [28,29]. ROS scavenging by histidine pretreatment resulted in a strong inhibition of the Ca^2+^ influx in both cell lines after 30 s CAP (*p* < 0.001 each, t-test with unpaired samples, Figure 3G,H). We tested whether the scavenging of reactive species could influence the CAP-induced acidification. PH measurements in ECS (#2) revealed no influence of cPTIO and histidine on the pH reduction by CAP, whereas the ^1^O_2_ scavenger sodium azide (NaN_3_) reduced the acidification process (Figure S3). In a previous study, we recognized that the species that were involved in CAP-induced Ca^2+^ influx were stable for at least one hour [26]. H_2_O_2_ can be stable in solution [30]; therefore, we tested whether the 30 s CAP-induced H_2_O_2_ amount (6 µM) changes the intracellular Ca^2+^ amount. The addition of H_2_O_2_ (in solution #4) on Mel Im and Mel Juso cells had no effects on the intracellular Ca^2+^ level (Figure S4). However, after preexposure of the cells to pH 6 in pbECS, H_2_O_2_ (#4, pH 6) caused a weak Ca^2+^ influx, with response rates of only 11% and 7% (Mel Im and Mel Juso, respectively, normalized to CAP).

### 2.5. An Acidic pH is Necessary For the Stability of Species That are Involved in the CAP-Induced Ca^2+^ Influx

**Figure 4:**
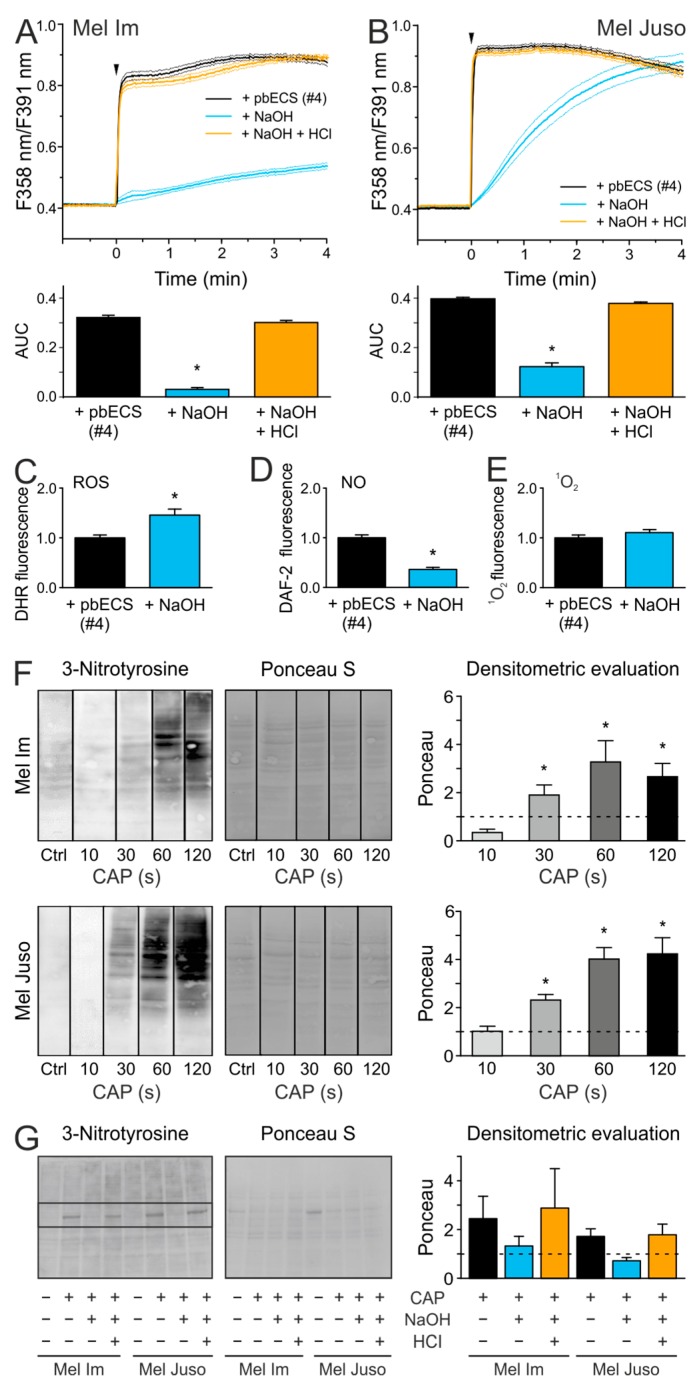
The stability of CAP-induced reactive species is pH dependent and NO is involved in protein nitration by CAP at an acidic pH. The acidic pH (5.8) of pbECS (#4) after CAP treatment was titrated to about 7.4 by NaOH (1 M) or further titrated back to a pH of about 6 using HCl (1 M). (**A**,**B**) PbECS (#4; 7 × 20 µL) treated with 120 s CAP was applied (arrow) to Mel Im ((**A**), *n* = 377–442) and Mel Juso ((**B**), *n* = 326–390) cells. (**C**–**E)** Fluorescence spectroscopic analysis of CAP-produced species in pbECS (#4; 7 × 20 µL) after pH titration, by using DHR 123 (**C**), (10 µM), DAF-2 (**D**), (10 µM), and Singlet Oxygen Sensor Green Reagent (E), (10 µM). The data are normalized to the respective controls (without pH titration). (**F**,**G**) Western Blot analysis of CAP-induced protein nitration after direct (**F**) and indirect (**G**) CAP-treatment of Mel Im and Mel Juso cells. Densitometric evaluation is shown as mean ± SEM. The pixel intensity of each lane is shown in Table S1.

We further wanted to figure out whether the acidic milieu is necessary for the stability of the relevant species of CAP-induced Ca^2+^ influx. The pH of a 120 s CAP-treated pbECS (#4) was titrated back to a physiological range and the addition of this solution onto melanoma cells caused a lower Ca^2+^ influx than the acidic pbECS (#4) solution (*p* < 0.001 each, t-test with unpaired samples, Figure 4A,B). Interestingly, a re-acidified 120 s CAP-treated pbECS (#4) solution induced a similar Ca^2+^ response as the non-titrated acidic pbECS (#4). The fluorescence measurements of the CAP-induced species production in pbECS (#4) showed that the pH titration resulted in an increased amount of ROS, a decreased amount of NO, and ^1^O_2_ production was not affected (*p* = 0.034, *p* = 0.026 and *p* = 0.22, respectively, *t*-test with paired samples, Figure 4C–E). The results suggest that, although NO alone seems to have no impact on the Ca^2+^ influx after direct CAP treatment, it could be a precursor of the relevant species. NO can react with superoxide (O_2_^•−^) to form the highly reactive peroxynitrite (ONOO^−^), which could induce tyrosine nitration. As shown by Western Blot analysis (Figure 4F), direct CAP treatment leads to a dose-dependent increase of 3-nitrotyrosine in Mel Im and Mel Juso cells (R = 0.65, *p* = 0.001, product-momentum correlation). Protein nitration was further observed after the application of a 120 s CAP-treated pbECS (#4) solution onto melanoma cells (*p* = 0.028, *n* = 3, Wilcoxon test, Figure 4G). The titration of the pH of 120 s CAP-treated pbECS (#4) to a physiological range inhibited CAP-induced 3-nitrotyrosine induction (*p* = 0.028, *n* = 3, Wilcoxon test), whereas protein nitration occurred after re-acidification of 120 s CAP-treated pbECS (#4, *p* = 0.046, *n* = 3, Wilcoxon test). The findings indicated that the CAP-induced acidification influences the CAP-induced RNS production and, consequently, protein nitration.

## 3. Discussion

The microenvironment of tumors is often acidic due to metabolic alterations, like upregulation of glycolysis and the pentose phosphate pathway, and an oxygen delivery that does not match the metabolic demand [31,32]. While the extracellular pH of most tissues is near 7.4, the tumor microenvironment can be in a range of pH 5.4–7.4 [33,34]. An extracellular pH of 6 triggers changes in cytoplasmatic Ca^2+^ level in cells of the skin, e.g., fibroblasts or endothelial cells, also in neuroblastoma cells, but not in the epidermoid carcinoma cell line A431 [35]. Surprisingly, a pH 4 did not induce a Ca^2+^ influx in melanoma cells in the present study, only with pH 3, an influx was observed, but this is a pH level that causes irreversible damage in e.g., erythrocytes and nerve cells [36,37]. This suggests that skin cancer cells have a remarkable pH resistance. Nevertheless, the CAP-induced Ca^2+^ influx in melanoma cells was higher at an acidic pH than under physiological conditions, which implies a synergistic mechanism of CAP-induced species and acidification.

**Figure 5:**
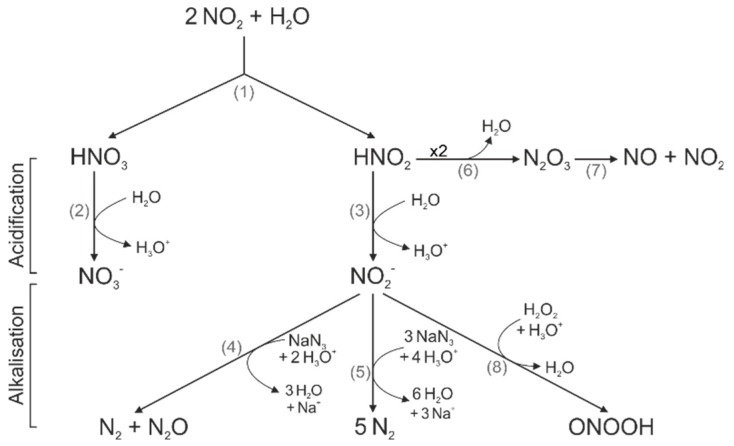
Schematic diagram of chemical species. The graphical reactions described in the text are represented to show acidification or consumption of acidic equivalents by NaN_3_ or generation of peroxynitrous acid (ONOOH).

We observed an acidification of solutions after CAP exposure, which is in line with results from several groups [38,39,40,41,42]. However, the cause of this acidification, as well as its impact on the antitumor properties of CAP, is not well understood. As observed here and reported previously, a longer CAP treatment duration caused a stronger acidification [38,39,40]. Thus, the concentration of CAP-induced reactive species seems to determine the level of acidification. Some groups assumed that H_2_O_2_, hydroperoxyl (HO_2_) or peroxynitrous acid (ONOOH) could be involved in the acidification process [38,39,43,44]. However, the most commonly suspected source of CAP-induced acidification was the production of nitric acid (HNO_3_) and nitrous acid (HNO_2_), which was due to hydrolysis of nitrogen dioxide (NO_2_) (1, see reaction numbers (grey) in Figure 5) [39,45,46,47]. The strong acid HNO_3_ (pK_a_ = −1.32) completely dissociates in water (2). The weak acid HNO_2_ (pK_a_ = 3.3) only dissociates partially and it is in an acid-base equilibrium with nitrite (NO_2_^−^) (3) [48,49]. In accordance to our observations, NaN_3_ was capable of reducing CAP-induced acidification in aqueous solutions [50]. This effect was attributed to a reaction of NO_2_^−^ (4,5) with NaN_3_, which consumes protons [49,50,51]. The underlying mechanism strengthened our assumption that HNO_2_ could be co-responsible for the CAP-induced acidification.

In the present study, the strongest CAP-induced acidification could be observed in bidestilled water. Treatment with other plasma devises also resulted in a stronger reduction in pH value in non-buffered solutions, like deionized water or NaCl, when compared to the buffered solutions [38,52]. CAP-induced acidification was dependent on the type of buffer. We assume that hydronium ions from reaction (2) and (3) were removed by the phosphate buffers. Therefore, pbECS (#4) and pbECS + HEPES (#5) only showed a slight pH decrease after CAP-treatment. In contrast, for a relevant reduction of CAP-induced acidification, a four-fold higher concentration of HEPES or MOPS was needed. Good buffers can be oxidized by H_2_O_2_; however, the process is slow and it only occurs at a concentration of at least 0.1 M H_2_O_2_ [53]. Nonetheless, a reaction of HEPES and MOPS with CAP-produced species might be a possible explanation for their poor CAP buffering.

We further revealed that the titration of 120 s CAP-treated pbECS (#4) to a physiological range reduces CAP-induced Ca^2+^ influx and protein nitration. Back-titration to a pH of around 6 resulted in a complete recovery of these effects. This suggests that the relevant species were only present under acidic conditions, but are not lost in temporary more alkalic conditions. Since the addition of NaOH to 120 s CAP-treated pbECS (#4) led to a reduction in NO formation, NO was one possible species being involved. The incorporation of a nitro group at the phenol ring of tyrosine can lead to several biochemical changes. For instance, a decrease in the pK_a_ value of the hydroxyl group, the addition of steric restrictions, or local conformational changes in the protein [54,55]. In consequence, a loss or gain of protein function or modulation of protein activity can be the result. A well-studied example for a protein inactivation due to nitration is the mitochondrial antioxidant manganese superoxide dismutase (MnSOD) [56,57]. In strong acidic solutions (pH ≈ 3.3), HNO_2_ decomposed to dinitrogen trioxide (N_2_O_3_) (6), which in turn reacts to NO and NO_2_ (7) [43,58,59].

It was shown that the HNO_2_-derived NO formation strongly depends on the pH value. Thereby, the amount of NO significant increased from pH 7.4 to pH 3 [60]. Furthermore, NO-induced nitration was described as a pH-dependent processes, with a maximum at ~pH 8 and low nitration levels at acidic conditions [61]. As in the present study, CAP-induced NO production in pbECS (#4), as well as CAP-induced 3-nitrotyrosine formation, showed a clear dose dependency; therefore, one can assume that the protein nitration by CAP was caused by HNO_2_-derived NO. However, NO does not seem to be responsible for the Ca^2+^ influx, as this was not altered by the NO scavenger cPTIO.

As observed by various groups, solutions that were strongly acidified by CAP showed stronger antibacterial effects than CAP-treated solutions that were closer to physiological pH [39,52,58,62]. Furthermore, an acidic pH alone did not have the same anti-bacterial effects as a CAP-treated solution of identical pH [39,58]. A synergetic effect of NO_2_^−^, H_2_O_2_, and acidic conditions was supposed to be responsible for the strong antibacterial effects of CAP [49,52,58]. One possible underlying mechanism was the production of the highly reactive ONOOH or ONOO^−^ (8) [52].

In contrast to that, the importance of the CAP-induced pH reduction on the anticancer effects of CAP was largely unexplored. It has been shown that a combination of H_2_O_2_ and HNO_2_ displayed antitumor effects that are comparable to CAP [63,64]. Thus, similar to the bacterial studies, a synergism of these species and an acidic pH could be also involved in the CAP effects on cancer cells. As observed by our group, CAP caused a Ca^2+^ influx in mouse fibroblasts, whereas the application of H_2_O_2_ at an equivalent concentration to CAP production (2.94 µM) had no effects [65]. In a previous study, we identified the ryanodine receptor (RyR) of the endoplasmic reticulum as one of the involved channels in CAP-induced Ca^2+^ influx [26]. H_2_O_2_ is a known inductor of the RyR opening, but concentrations between 100 µM and 10 mM were necessary [66,67]. Although the H_2_O_2_ concentration was too low for triggering Ca^2+^ influx alone, it could still be involved in the CAP-induced effects on cancer cells. The fact that pretreatment of the melanoma cells with histidine, which scavenges H_2_O_2_, caused a significant reduction of CAP-induced cytoplasmic Ca^2+^ elevation supports this theory. In addition, the incubation of the cells with pH 6 increased the sensitivity of melanoma cells to H_2_O_2_, whereby a Ca^2+^ influx in 10% of Mel Im and 7% of Mel Juso cells could be observed. The differential composition of routinely used media causes substantial differences in CAP-induced chemicals, e.g., H_2_O_2_ is markedly different between DMEM and RPMI. The manuscript used mainly minimal self-composed media to investigate particular components, as these have many ingredients, e.g., to support that glucose has no impact on CAP-induced species production.

In summary, we demonstrated the importance of CAP-induced acidification in CAP-induced intracellular Ca^2+^ elevation in malignant melanoma cells. The inhibition of acidification by stronger buffer systems reduced the CAP-induced Ca^2+^ influx. HNO_3_ and HNO_2_, which are produced by hydrolysis of CAP-induced NO_2_, are hypothesized to cause this acidification, as we could demonstrate a pH-dependent CAP-induced NO-production and protein nitration. The scavenger experiments revealed that ROS were equally involved in CAP-induced Ca^2+^ influx, which suggests a synergistic effect of CAP-induced ROS, RNS, and acidic conditions. The CAP generates a complex cocktail of unknown recipe, of which some essential ingredients were investigated; nevertheless, this will help to develop CAP as a possible cancer therapy. CAP treatment could be performed e.g., after the surgical removal of the tumor. The acidic conditions in tumors could support the CAP-effects and contribute to cancer cell-specificity. 

## 4. Materials and Methods

### 4.1. Cell Culture

The Mel Juso cell line (DSMZ: ACC74) was established from a primary cutaneous melanoma and the Mel Im cell line, as obtained from Prof. Dr. Judith Johnson [68], was descended from a metastatic malignant melanoma. The DSMZ recently authenticated the cell lines. The cells were cultivated in RPMI 1640 (Mel Juso) and in Dulbecco’s modified eagle’s medium (DMEM, Mel Im) bought from Sigma-Aldrich, Steinheim, Germany, as described previously [69]. Mycoplasma contamination is excluded on a regular basis for both cell lines.

### 4.2. Plasma Device

The experiments were performed with a miniFlatPlaSter plasma device, which was developed by the Max Planck Institute for Extraterrestrial Physics (Garching, now Terraplasma GmbH, Garching, Germany) for treating tumor cells and tissue. The plasma is generated from ambient air using the SMD technology [2,5]. Technical details of the device have recently been published [70].

### 4.3. Chemicals and Solutions

The ECS, the pbECS, and further variations in solutions used in Ca^2+^ imaging experiments were freshly prepared, and Table 1 lists the components. The Ponceaus S solution was composed of 0.2% Ponceau S, 3% trichloroacedic acid, and 3% sulfosalicylic acid. Sources for chemicals and solutions: Dulbecco’s phosphate buffered saline (DPBS) (Sigma-Aldrich, Steinheim, Germany), fura-2 AM and pluronic F-127 (Biotium, Fremont, CA, USA), ionomycin (2 µM, Enzo Life Sciences, Farmingdale, NY, USA), DMSO (Sigma-Aldrich), histidine (3 µM, Sigma-Aldrich), NaN_3_ (Sigma-Aldrich), 30% (w/w) H_2_O_2_ (6 µM, Sigma-Aldrich), cPTIO (250 µM, Cayman Chemical, Ann Arbor, MI, US), anti-nitrotyrosine antibody (MERCK, Darmstadt, Germany, 06-284), and alkaline phosphatase-conjugated rabbit secondary antibody (Cell Signaling, Danvers, MA, USA, 7054S). Dihydrorhodamine 123 (DHR 123) and the Fluorimetic Hydrogen Peroxide Assay Kit both from Sigma-Aldrich, 4,5-diaminofluorescein (DAF-2) from Enzo Life Sciences (Farmingdale, NY, USA) and the Singlet Oxygen Sensor Green Reagent purchased from Molecular Probes (Eugene, OR, USA) were used to perform the fluorescence spectroscopic analyses of CAP-induced reactive species. 

### 4.4. Ca^2+^ Imaging

The experimental approach has been performed, as previously described [26]. CAP treatment was executed in two ways: direct or indirect. The experimental set up was shown in a previous publication [26]. The only modification of the indirect CAP treatment method was a volume increase from 120 µL to 140 µL of the CAP-exposed solution, being deposited in seven separate 20 µL droplets. As before, 100 µL of the CAP-exposed solution was added to these cells.

For application experiments without CAP, the cells were washed in pbECS with pH 7.4, unless stated otherwise. For titration experiments, seven separate 20 µL drops of pbECS in a six-well plate without cells were exposed to 120 s CAP and transferred into a 1.5 mL reaction vessel. Immediately, 1 µL pbECS or 1 µL NaOH (1 M) or 1 µL NaOH and 1 µL HCl (both 1 M) were added and mixed by vortexing. The test solution or titrated pbECS were applied into an aluminum ring of 6.5 mm diameter after 60 s of Ca^2+^ measurement.

The experimental setup and procedures were described in detail [71]. In brief, the fluorescence of fura-2-stained single cells was recorded while the sample was alternatingly excited at 358 nm and 391 nm with a frequency of 1 Hz with a Polychrome V monochromator (Till Photonics, München, Germany). A cooled charge-coupled device (CCD) camera collected a fluorescence emission above 440 nm. The area under the curve (AUC) of the fluorescent time course of individual cells was calculated for a period of 120 s after stimulation by CAP treatment or a solution, the 10 s before this stimulation served as reference.

### 4.5. Fluorescence Spectroscopic Detection of Reactive Species

The fluorescence dyes DHR 123 (ROS-indicator), DAF-2 (NO-indicator), and the Singlet Oxygen Sensor Green Reagent were dissolved in the solution to be analyzed at a concentration of 10 µM. In a six-well plate (Corning Incorporated, Corning, NY, USA), seven separate 20 µL drops of the respective solution were exposed to different CAP doses or remained untreated. Immediately after CAP treatment, 100 µL of the solution was added to a black 96-well plate (Corning Incorporated) and the fluorescence was measured with the CLARIOstar ELISA-Reader (BMG LABTECH, Ortenberg, Germany) at Ex = 500 ± 8 nm und Em = 530 ± 8 nm. H_2_O_2_ concentration was determined by using the Fluorimetic Hydrogen Peroxide Assay Kit. The analyzed solutions were added to a six-well plate in 7 × 20 µL drops and they were treated or not treated with CAP. A volume of 50 µL of the CAP-exposed solution was transferred into a black 96-well plate. The further procedure was performed according to the manufactures protocol in duplicates. The fluorescence of the peroxidase substrate was measured at Ex = 540 nm and Em = 590 nm by using the CLARIOstar ELISA-Reader.

### 4.6. Western Blot Analysis

On the day before CAP treatment, 200,000 cells per six-well plate (direct CAP treatment, double well), or 50,000 cells per 24-well plate (indirect CAP treatment, four wells) were seeded. For titration experiments with indirect CAP treatment, seven separate 20 µL drops of pbECS were exposed to 120 s CAP and titrated with NaOH or HCl, as described under Ca^2+^ imaging. After a washing step with pbECS, the whole CAP-treated solution was applied onto the cells. Direct CAP treatment was only performed with a residual fluid (33 ± 1 μm fluid layer height) of DMEM or RPMI on the cells. Immediately thereafter, the cells were covered with 2 mL medium. After direct and indirect CAP treatment, followed an incubation of one hour at 37 °C and 8% CO_2_. The radioimmunoprecipitation assay (RIPA) buffer was used to produce total protein lysates, as described previously [72]. The RIPA protein lysates were separated via sodium dodecyl sulphate polyacrylamide gel electrophoresis on a 10% gel and blotted on a poly-vinylidene difluoride (PVDF) membrane. For Ponceau staining, the membrane was briefly activated with pure methanol and then washed with bidestilled water. The membrane was incubated for 15 min. in a 0.2% Ponceau S solution and the excess dye was carefully removed with bidestilled water. The Ponceau staining was captured with a Perfection V800 scanner (Seiko Epson Corporation, Suwa, Japan). After another washing step with bidestilled water, the membrane was incubated for one hour in the blocking buffer containing 5% milk powder and Tris-buffered saline with 0.1% tween (TBS-T). The membrane was incubated over night with the primary anti-nitrotyrosine antibody (1:1000, MERCK, 06-284), in TBS-T, followed by an incubation of 1 h at room temperature with the alkaline phosphatase-conjugated rabbit secondary antibody (Cell Signaling, 7054S) 1:3000 in TBS-T. The Claity™ Western ECL Substrate was used to perform protein visualization (Bio-Rad, Hercules, CA, USA).

### 4.7. pH Measurement

For pH measurement, 7 × 20 µL drops of the respective solution in 35 mm diameter tissue culture dishes (Sarstedt, Nümbrecht, Germany) were exposed to CAP and then transferred into a 1.5 mL reaction vessel. The pH measurement was performed immediately by using the LAQUAtwin pH meter (HORIBA Scientific, Kyōto, Japan). 

### 4.8. Statistical Analysis 

Two groups with at least ten samples were compared with a paired or unpaired t-test, and smaller independent groups with a U-test. Repeated measurements and multiple groups were compared by ANOVA and HSD post-hoc tests. The parametric association between variables was quantified by a product-momentum correlation. Statistica 8 (Statsoft, Tulsa, OK, USA) or GraphPad Prism 5 (GraphPad Software Inc., San Diego, CA, USA) were used for statistical analysis. Time courses and AUCs are presented with mean and 99% confidence intervals, other results with mean ± SEM, except for; *p* < 0.05 was considered to be significant. 

## 5. Conclusions

We could show in this study that a synergistic effect of CAP-induced ROS, RNS and acidic conditions affect the intracellular Ca^2+^ homeostasis of melanoma cells. As the microenvironment of tumors is often acidic, this might be one reason for the specific anti-cancer effects of CAP.

## Figures and Tables

**Table 1:** Composition of the used solutions with concentrations in mM and an assigned solution number used throughout the manuscript.

	Solution	PBS	ECS HEPES	ECS HEPES − Glucose	pbECS	pbECS + HEPES	ECS MOPS	pbECS − Ca^2+^	pbECS − Ca^2+^ 3 × Buffered
(M)		#1	#2	#3	#4	#5	#6	#7	#8
**NaCl**		137.9	145.0	145.0	133.0	133.0	145.0	133.0	108.8
**KCl**		2.5	5.0	5.0	3.5	3.5	5.0	3.5	0.6
**Glucose**			10.0		10.0	10.0	10.0	10.0	10.0
**KH_2_PO_4_**		2.5			1.5	1.5		1.5	4.4
**Na_2_HPO_4_**		7.0			8.1	8.1		8.1	24.2
**CaCl_2_**		1.3	1.3	1.3	1.3	1.3	1.3		
**MgCl_2_**		1.0	1.0	1.0	1.0	1.0	1.0	1.0	1.0
**EGTA**								10.0	10.0
**HEPES**			10.0	10.0		10.0			
**MOPS**							10.0		

EGTA (ethylene glycol-bis(β-aminoethyl ether)-*N*,*N*,*N*′,*N*′-tetraacetic acid), MOPS (3-(*N*-morpholino)propanesulfonic acid).

## Supplementary Materials

The following are available online at https://www.mdpi.com/2072-6694/11/5/671/s1, Figure S1: The CAP-induced intracellular Ca^2+^ influx is pH dependent. Figure S2: CAP-produced reactive species vary in different media, Figure S3: The CAP-induced acidification could be inhibited by NaN_3_, Figure S4: Application of H_2_O_2_ onto melanoma cells causes a minor Ca^2+^ influx, Figure S5: Whole blots of the Western Blot analysis of CAP-induced protein nitration after direct CAP treatment of Mel Im and Mel Juso cells, shown in Figure 4F, Table S1: Densitometric evaluation of the Western Blot analysis shown in Figure 4F,G.
