# Supplementary material for: Acidification is an Essential Process of Cold Atmospheric Plasma and Promotes the Anti-Cancer Effect on Malignant Melanoma Cells
Source: Cancers (Basel). 2019 May 14;11(5):671. doi: 10.3390/cancers11050671 (PMC6562457; doi:10.3390/cancers11050671)
Supplement: Supplementary file 1 [file cancers-11-00671-s001.pdf]

cancers

Article

Acidification is an Essential Process of Cold Atmospheric Plasma and Promotes the Anti-Cancer Effect on Malignant Melanoma Cells

Christin Schneider, Lisa Gebhardt, Stephanie Arndt, Sigrid Karrer, Julia L. Zimmermann, Michael J.M. Fischer and Anja-Katrin Bosserhoff

Supplementary Material

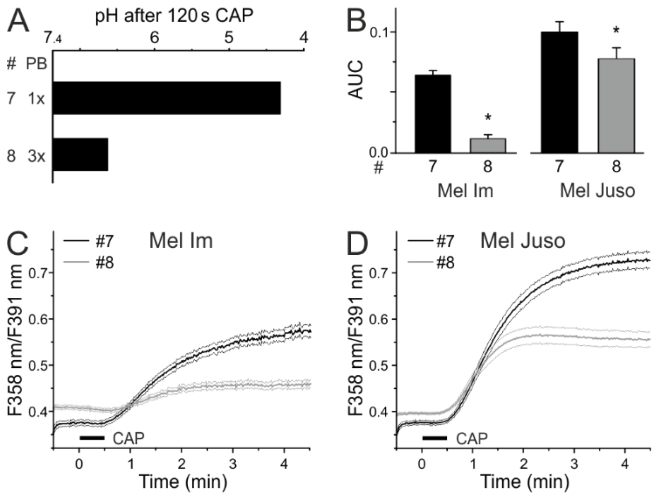

Figure S1. The CAP-induced intracellular Ca^{2+} influx is pH dependent. (**A**) PH measurement of 120 s CAP treated pbECS – Ca^{2+} (#7) and 3x buffered pbECS – Ca^{2+} (#8). (**B**–**D**) Analysis of 30 s CAP-induced Ca^{2+} influx in Mel Im ((**B**–**C**), *n* = 225–464) and Mel Juso ((**B**,**D**), *n* = 357–427) cells by using pbECS – Ca^{2+} (#7) and 3x buffered pbECS – Ca^{2+} (#8).

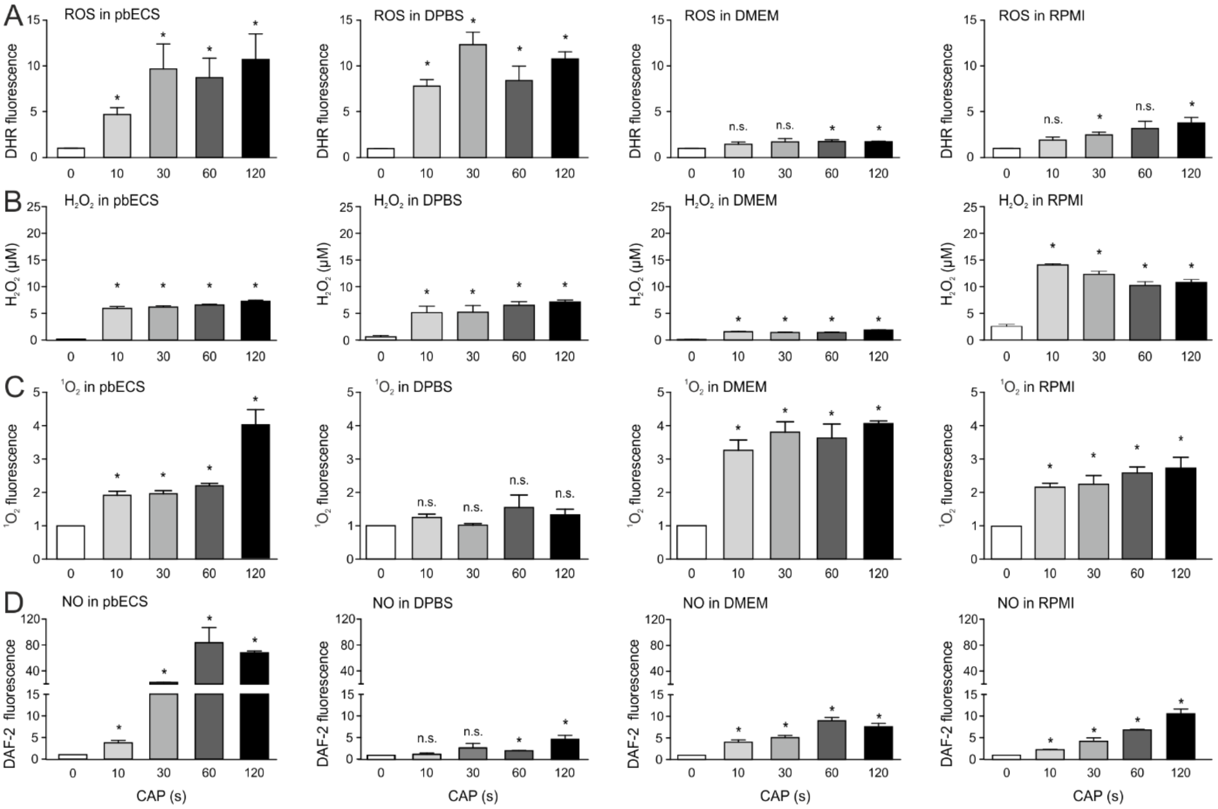

Figure S2. CAP-produced reactive species vary in different media. (**A**) Fluorescence spectroscopic analysis of CAP-induced species production in 7 × 20 µL drops of pbECS (#4), DPBS, DMEM and RPMI, by using DHR 123 (**A**), Fluorimetic Hydrogen Peroxide Assay Kit (**B**), Singlet Oxygen Sensor Green Reagent (**C**) and DAF-2 (**D**). Data are mean ±SEM.

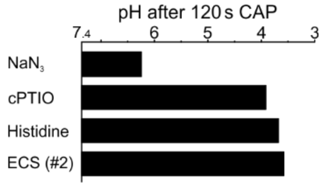

Figure S3. The CAP-induced acidification could be inhibited by NaN_3. PH measurement of ECS (#2, 7 × 20 µL drops) solutions, supplemented with histidine (3 mM), cPTIO (100 µM) or NaN_3 (5 mM), after 120 s CAP-treatment.

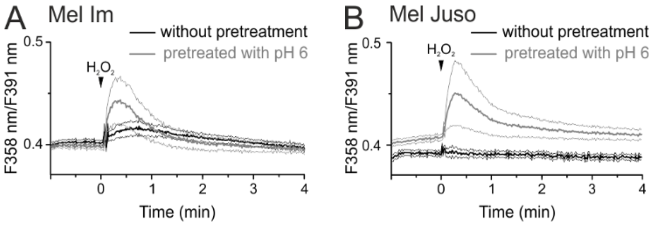

Figure S4. Application of H_2O_2 onto melanoma cells causes a minor Ca^{2+} influx. H_2O_2 (6 µM), solved in pbECS (#4) pH 6 or pH 7.4, was added onto Mel Im ((**A**), *n* = 334–343) and Mel Juso ((**B**), *n* = 299–370) cells, which were pretreated for 5 min with the same solution without H_2O_2.

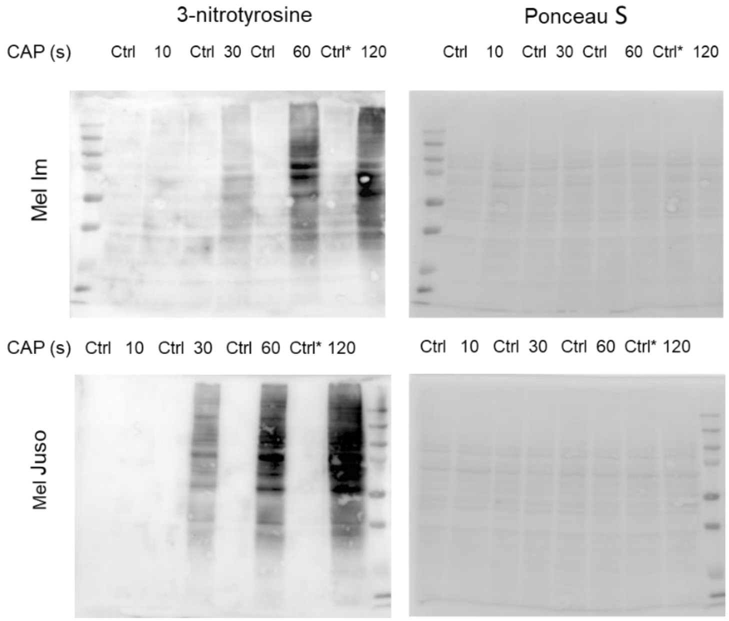

Figure S5. Whole blots of the Western Blot analysis of CAP-induced protein nitration after direct CAP treatment of Mel Im and Mel Juso cells, shown in Figure 4F. Ctrl* (120 s without medium) was used for the densitometric measurement.

Table 1. Densitometric evaluation of the Western Blot analysis shown in Figure 4F,G. Values represent the mean pixel intensity per lane (*n* = 3, arbitrary unit, values × 10^6).

**Figure 4F Pixel Intensity**	**Ctrl***	**10 s CAP**	**30 s CAP**	**60 s CAP**	**120 s CAP**
**Mel Im**					
**3-nitrotyrosine**	14	6	32	52	43
**Poncau S**	23	24	26	26	26
**Mel Juso**					
**3-nitrotyrosine**	13	12	29	48	53
**Poncau S**	20	20	20	19	20
**Figure 4G Pixel Intensity**	**Ctrl**	**120 s CAP**	**120 s CAP + NaOH**	**120 s CAP + NaOH + HCl**	
**Mel Im**					
**3-nitrotyrosine**	14	24	16	30	
**Poncau S**	64	56	69	69	
**Mel Juso**					
**3-nitrotyrosine**	17	25	14	28	
**Poncau S**	81	73	72	82	

© 2019 by the authors. Licensee MDPI, Basel, Switzerland. This article is an open access article distributed under the terms and conditions of the Creative Commons Attribution (CC BY) license (http://creativecommons.org/licenses/by/4.0/).
